# Increased Neural Activity of a Mushroom Body Neuron Subtype in the Brains of Forager Honeybees

**DOI:** 10.1371/journal.pone.0000371

**Published:** 2007-04-18

**Authors:** Taketoshi Kiya, Takekazu Kunieda, Takeo Kubo

**Affiliations:** Department of Biological Sciences, Graduate School of Science, The University of Tokyo, Tokyo, Japan; Centre de Recherches su la Cognition Animale-Centre National de la Recherche Scientifique and Université Paul Sabatier, France

## Abstract

Honeybees organize a sophisticated society, and the workers transmit information about the location of food sources using a symbolic dance, known as ‘dance communication’. Recent studies indicate that workers integrate sensory information during foraging flight for dance communication. The neural mechanisms that account for this remarkable ability are, however, unknown. In the present study, we established a novel method to visualize neural activity in the honeybee brain using a novel immediate early gene, *kakusei*, as a marker of neural activity. The *kakusei* transcript was localized in the nuclei of brain neurons and did not encode an open reading frame, suggesting that it functions as a non-coding nuclear RNA. Using this method, we show that neural activity of a mushroom body neuron subtype, the small-type Kenyon cells, is prominently increased in the brains of dancer and forager honeybees. In contrast, the neural activity of the two mushroom body neuron subtypes, the small-and large-type Kenyon cells, is increased in the brains of re-orienting workers, which memorize their hive location during re-orienting flights. These findings demonstrate that the small-type Kenyon cell-preferential activity is associated with foraging behavior, suggesting its involvement in information integration during foraging flight, which is an essential basis for dance communication.

## Introduction

A variety of animals, from nematode to human, show social behavior [1], [2]. The social behaviors allow for individuals to create an entity greater than the sum of the individuals and provide the key to successful adaptation to the environment. One of the most striking features of the highly-ordered animal society is the ability to share information among individuals. In turn, higher communicative ability is a fundamental basis that enables animals to maintain a more evolved society [3].

Honeybees (*Apis mellifera* L.) organize a highly-ordered society and have a sophisticated communicative ability known as the ‘dance communication’ [4]–[7]. Worker honeybees that find a rich food source return to the hive and might transmit the information on the location of the food source to their nestmates using a symbolic dance. The dance information is decoded into the spatial information of the food source by the other worker bees (followers) that follow the dancers [4]–[7]. During foraging flights, worker honeybees integrate the incoming sensory information: they estimate the distance of food sources based on the amount of optic flow they perceive, and direction based on the position of the sun [5], [8], [9], which are the essential bases for the expression of dance communication. Although there is a considerable amount of research concerning the sensory basis of these remarkable abilities [8]–[11], almost nothing is known about the underlying neural mechanisms.

As a first step in elucidating the neural mechanisms of these remarkable abilities, it is important to identify active brain regions in dancing and foraging honeybees that might be involved in dance communication and/or information integration during foraging flight. Although methods to detect the expression of immediate early genes (IEGs) as markers of neural activity are widely used in vertebrates [12]–[15], neural IEGs have not yet been identified in insects. In the present study, we identified a novel IEG that can be used as a neural activity marker and found that the neural activity of a mushroom body (MB) neuron subtype is preferentially increased in foraging honeybees, suggesting its involvement in information integration during foraging flight.

## Results

### A novel non-coding IEG, *kakusei*, can be used as a marker to visualize neural activity in the honeybee brain

**Figure 1:**
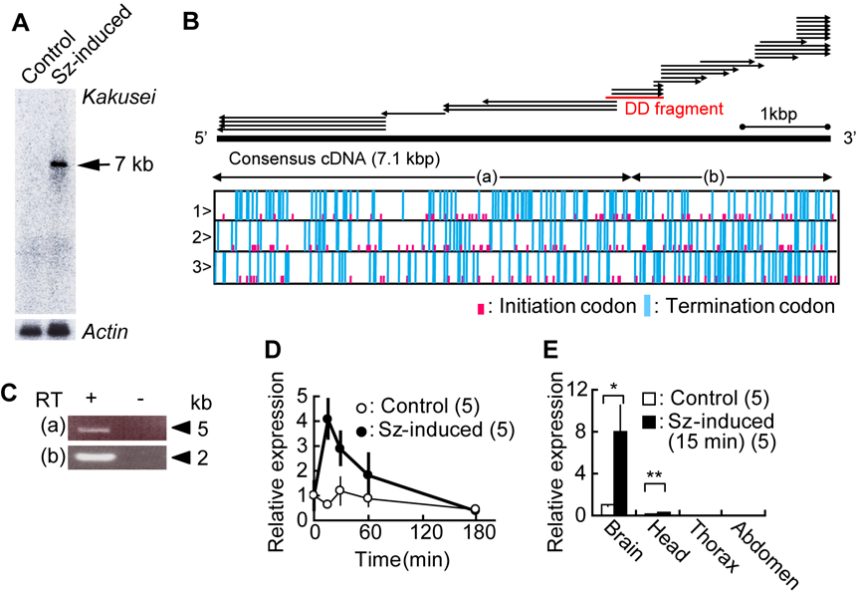
Identification and characterization of a novel non-coding immediate early gene, *kakusei*. (A) Northern blot analysis was performed using RNAs extracted from the brains of workers collected 15 min after seizure induction (Sz-induced lane) and workers anesthetized with CO_2_ for 15 min (Control lane). Using the same membrane, *actin* was detected as a loading control. The approximately 7-kb long signal is indicated by a triangle. (B) Overview of *kakusei* cDNA contig and open reading frame analysis. Arrows indicate cDNA subclones identified by 5′-and 3′-rapid amplification of cDNA ends (RACE) methods. The thin red bar in the middle of the arrows shows the DNA fragment identified by differential display screening (DD fragment). The solid bar indicates the full-length consensus *kakusei* cDNA. Lower box indicates open reading frame analysis in each reading frame of the consensus *kakusei* DNA. Note the lack of significantly long open reading frames. (C) Actual expression of the assembled cDNA sequence was confirmed by RT-PCR using primers designed to amplify the regions shown in (B). Bands of the expected size were detected for both regions (a) and (b), and the sequence was the same as the consensus sequence. Experiments were repeated five times, and performed with (+) or without (−) the RT (reverse transcriptase) reaction, confirming that there is no genomic DNA contamination in the samples. (D) Time course of *kakusei* expression investigated by real-time RT-PCR. (E) *Kakusei* expression in various body parts investigated by real-time RT-PCR (*, *P* = 0.0487; **, *P* = 0.0036; Student's *t*-test). All data are shown as the means±SEM.

To identify IEGs, we used the differential display method to search for honeybee genes that are immediately induced in the brain by neural activity. To evoke strong neural activity in the brain, seizures were induced by awakening workers from ice-cold induced anesthesia, because some of the IEGs were identified by inducing seizures in the animals [16], [17]. When the workers are awoken from anesthesia, they show seizure-like movement with their legs and body shaking. Using differential display screening of approximately 6500 bands, which were derived from mRNAs extracted from the brains of seizure-induced and non-treated bees, 49 candidate bands were identified. Among them, we selected nine candidates that showed a pronounced difference in band intensity between the seizure-induced and non-treated bees. After preliminary Reverse transcription-polymerase chain reaction (RT-PCR) analysis of these candidates, we finally focused on a single candidate that showed the most prominent and reproducible seizure-induced transcript increase. As a result, we identified a novel IEG that we named *kakusei* after the word ‘awakening’ in Japanese (the whole sequence of *kakusei* was deposited as DDBJ accession number AB252862). To examine the size of the *kakusei* transcript, we performed Northern blot analysis using total RNA isolated from whole brains of bees anesthetized with CO_2_ and bees awakened from CO_2_-induced anesthesia. The results indicated that the induced *kakusei* transcript was approximately 7 kb long (Figure 1A). There was no significant open reading frame in any of the three possible reading frames of the *kakusei* cDNA sequence, suggesting that the *kakusei* transcript functions as a non-coding RNA (Figure 1B). RT-PCR experiments and sequence analysis also confirmed that the contig *kakusei* sequence is expressed as continuous transcripts (Figure 1C).

**Figure 2:**
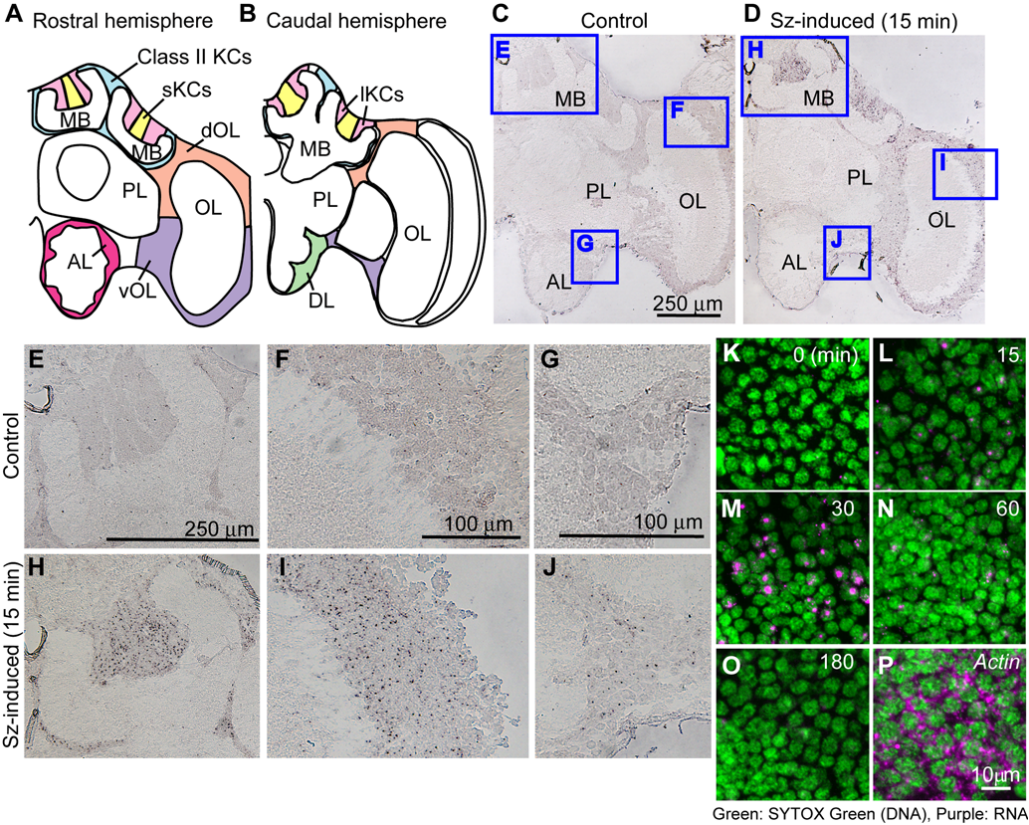
*Kakusei* can be used as a marker to visualize neural activity in the honeybee brain. (A and B) Schematic drawings of the rostral (A) and caudal (B) brain hemisphere of worker honeybees. Positions of neuron somata are shown in color. (C–J) Expression of *kakusei* was detected by *in situ* hybridization using coronal brain sections of control (C, E–G) or seizure-induced (Sz-induced) (D, H–J) bees. Areas corresponding to panels (E–J) are boxed in (C) and (D). (K–O) Subcellular localization of *kakusei* was investigated by fluorescent *in situ* hybridization. Nuclei were visualized by SYTOX Green, which stains DNA. Numbers of each picture indicate time after seizure. *Actin* mRNA was detected as a control that is translated into protein in the cytoplasm (P). AL, antennal lobe; Class II KCs, class II Kenyon cells; DL, dorsal lobe; dOL, dorsal OL; lKCs, large-type Kenyon cells; MB, mushroom body; OL, optic lobe; PL, protocerebral lobe; sKCs, small-type Kenyon cells; vOL, ventral OL.

*Kakusei* expression was transiently induced in the whole brain after awakening the workers from anesthesia induced by either CO_2_ [Figure 1D; Sz (seizure)-induced] or ice-cold (data not shown). Real-time RT-PCR revealed that *kakusei* is expressed predominantly in the brain, suggesting a brain-specific function (Figure 1E). *In situ* hybridization revealed that *kakusei* expression can be detected in every brain region, including the MBs, optic lobes (OLs), and antennal lobes (ALs) in a seizure induction-dependent manner (Figure 2C–J), suggesting that *kakusei* can be used as a marker in broad brain regions. In addition, *kakusei* signals (purple) were detected exclusively in the nuclei (green) of brain neurons (Figure 2K–O), reflecting the characteristics of the *kakusei* transcript as a non-coding RNA. This notion was clearly demonstrated when the *kakusei* transcript localization was compared to that of *actin*, which is transported to the cytoplasm to be translated into protein and is detected as a broadly-distributed signal in the cytoplasm (Figure 2P). This characteristic *kakusei* signal staining enabled us to count and quantify the number of *kakusei*-positive neurons.

We next examined whether *kakusei* expression reflects physiological neural activity by testing whether *kakusei* expression was induced in the brain upon light exposure. First, workers were dark-adapted by keeping them in the dark overnight. The next day, experimental bees (light-exposed) were exposed to white light for 30 min, and then used for *in situ* hybridization. Dark-adapted bees were used as a control. *Kakusei* expression was detected in the OL and MB neurons of the light-exposed bees (Figure S1A–F). The expression of *kakusei* was particularly prominent in the lamina neurons, which receive direct input from retinal neurons (Figure S1C and D). In contrast, there was no strong *kakusei* expression in the AL neurons (Figure S1G and H). These results indicate that *kakusei* can be used as a marker to visualize physiological neural activity in the honeybee brain.

**Figure 3:**
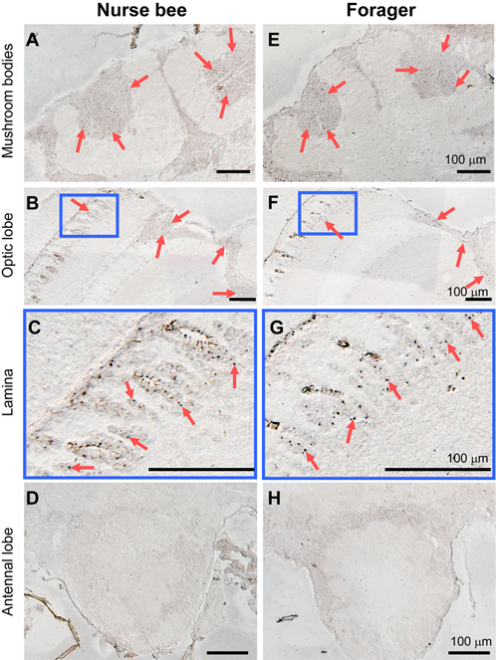
*Kakusei* expression in the brains of bees that exhibited phototaxis. *In situ* hybridization was performed using thin sections (10 µm) of the brains of nurse bees that did not move to the light side (N = 4, A–D) and foragers that had phototactic behavior (N = 5, E–H). (C and G) Magnified views of boxed regions in panels (B) and (F), respectively. Bars indicate 100 µm. Arrows indicate *kakusei*-positive cells. *Kakusei* expression was detected both in the mushroom bodies (A and E) and optic lobes (B, C, F and G) of nurse bees (A–C) and foragers (E–G), and not in the antennal lobes (D and H).

To further examine whether *kakusei* expression can be detected in the brains of bees that exhibit physiologic behaviors, we studied *kakusei* expression in the brains of bees exhibiting phototactic behavior. According to a previous report [18], we collected foragers that moved to the lighted side and nurse bees that did not move to the lighted side 30 min after the start of the phototactic behavior, and investigated *kakusei* expression in the brains (see Figure S2 for the experimental procedures). Almost the same *kakusei* expression pattern was observed in the brains of both the foragers and nurse bees: a large number of neurons in the optic lobes were *kakusei*-positive (Figure 3), which was similar to the findings in the light-exposed bees (Figure S1). These results suggest that *kakusei* expression is not so sensitive as to be induced by the neural activity specific to phototactic behavior, and that the activity in the optic lobes, which could be induced by visual inputs upon light illumination, is predominant even in the brains of bees that exhibited phototaxis. The fact that *kakusei* expression was observed in the optic lobes of bees that were not dark-adapted and had natural phototactic behavior strongly suggests that *kakusei* expression reflects neural activity under normal physiologic conditions, although we still cannot exclude the possibility that light-exposure is a stressor to the honeybee.

### Neural activity is increased in the small-type Kenyon cells of the dancer brain

**Figure 4:**
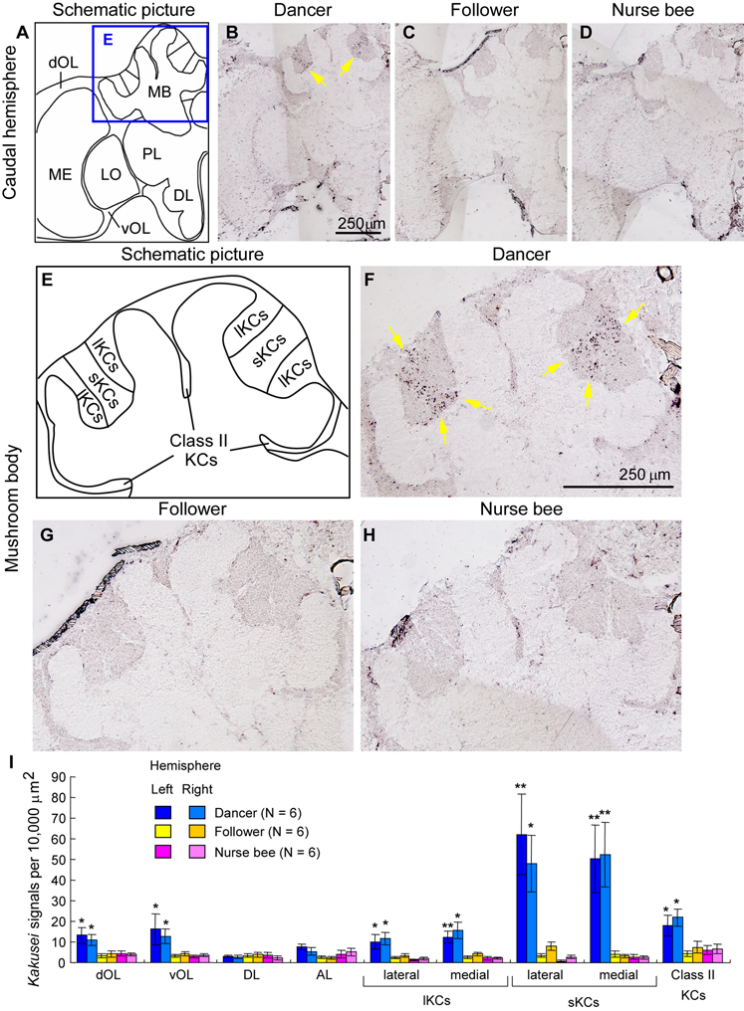
Neural activity of the sKCs is prominently increased in the dancer brain. (A and E) Schematic drawings of the caudal hemisphere (A) and MB (E). (B–D and F–H) *In situ* hybridization of *kakusei* in the dancer (B and F), follower (C and G), or nurse bee (D and H) brains. (I) Quantification of *kakusei*-positive cells in various brain regions. Asterisks indicate significant difference from other behavioral groups (*, *P*<0.05; **, *P*<0.01; Tukey-Kramer's test after ANOVA). Arrows indicate the *kakusei*-expressing neurons.

The workers shift their tasks from nursing their brood (nurse bees) to foraging for nectar and pollen (foragers) according to the number of days after eclosion [4], [7]. When foragers successfully find food sources, some of them perform a dance to enroll followers to forage [7]. Therefore, we next examined *kakusei* expression in the brains of the dancers, followers, and nurse bees to identify the brain regions involved in dance communication (Figure 4). The individual workers were caught immediately after confirming their behaviors in the observation hives, and used for *in situ* hybridization. The bees caught from the observation hives were immediately anesthetized by ice-cold water and kept on ice until use for *in situ* hybridization to maintain the current state of the *kakusei* transcripts in the brain. There was a characteristic *kakusei* expression pattern in the dancer brains, especially in the MBs (Figure 4B). The honeybee MBs consist of three types of intrinsic neurons termed large-type Kenyon cells (lKCs), small-type KCs (sKCs), and class II KCs (Figure 4E) [19]–[22]. The most prominent *kakusei* expression was observed in the sKCs (Figure 4B and F), whose somata are located in the center of the MBs (Figure 4A and E). In contrast, only a small number of positive cells were detected in the brains of followers and nurse bees (Figure 4C, D, G, and H). *Kakusei* expression in the central complex neurons was not clear, as we could not identify the central complex neurons in our *in situ* hybridization experiments.

Quantitative analysis revealed that the number of *kakusei*-positive cells in the sKCs was approximately 20 times higher in the dancers than in the followers or nurse bees (Figure 4I). In addition, *kakusei* expression was also weakly induced in the lKCs and class II KCs, as well as in the dorsal and ventral OL neurons (dOL and vOL, respectively) (Figure 4I). A three-factor ANOVA [F1: bee type; F2: brain region (repeated measure); F3: brain hemisphere (repeated measure)] revealed that there was a significant difference between the bee type and brain region (F1 and F2: *P*<0.0001, respectively; significant interaction between F1 and F2: *P*<0.0001). In contrast, there was no significant difference between the right and left hemispheres (F3: *P* = 0.9455). Significant differences between bee types were observed in the dOL, vOL, lKCs, sKCs, and class II KCs (Figure 4I; *, *P*<0.05; **, *P*<0.01; Tukey-Kramer's test). These results indicate that the neural activity in these brain regions, especially the sKCs, is increased in the dancer brain. The differences in *kakusei* expression between dancers and followers could not be due to their different developmental stages but only to their behavioral differences, because both the dancers and followers are thought to be of the same behavioral stage [5], [7]. Thus, the prominent neural activity in the sKCs observed in the dancer brains is likely due to their characteristic behaviors.

### Neural activity of the small-type Kenyon cells is also increased in the forager brain

According to the expression profile (Figure 1D and Figure 2K–O), *kakusei* expression reflects neural activity that occurred 15 to 60 min prior to sampling of the bees. In our observation, dancers repeated the dance every 5 to 7 min and the foraging every 10 to 15 min. Thus, the neural activity detected in the dancer brains might be due not only to the dancing behavior, but also to the preceding foraging behavior. To address this question, we examined *kakusei* expression in the brains of foragers. Only some of the foragers that succeed in finding food display dance behavior [7], [23]. Thus, when we analyze foragers, only some of them are expected to be dancers.

**Figure 5:**
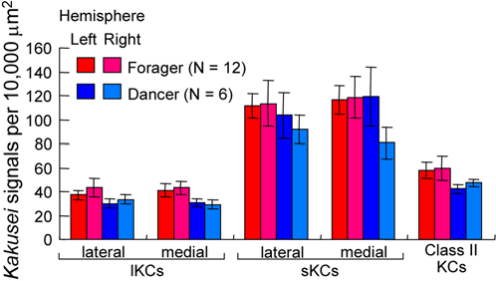
Foragers showed similar *kakusei* expression pattern to the dancers. Quantification of *kakusei* expression in the brains of foragers and dancers. Foragers also showed the sKC-preferential *kakusei* expression pattern. There was no significant difference in the number of *kakusei*-positive cells (*P*>0.05; two-factor ANOVA). Note that we repeated the experiments for the ‘dancer’ group here, and bees used in the ‘dancer’ group for this experiment were different from those used in the previous experiment (Figure 4).

Therefore, we collected foragers with pollen loads (an indication that they were successful in finding food) in front of the hive entrance before we checked whether or not they danced in the hive. The results indicated that every forager (N = 12) had a *kakusei* expression pattern similar to that of the dancers (N = 6). In addition, there was no significant difference in the density of *kakusei*-positive cells in the MB neurons, including in the sKCs, between these bees [Figure 5; *P*>0.05, two-factor ANOVA (F1: bee type; F2: brain hemisphere)]. Thus, these results suggest that the increased sKC neural activity in the dancer brain is associated with foraging behavior rather than dancing behavior, although we cannot exclude the possibility that the foragers we examined also exhibited the dance behavior shortly before the observation period.

### Re-orienting bees showed different *kakusei* expression patterns from foragers and dancers

**Figure 6:**
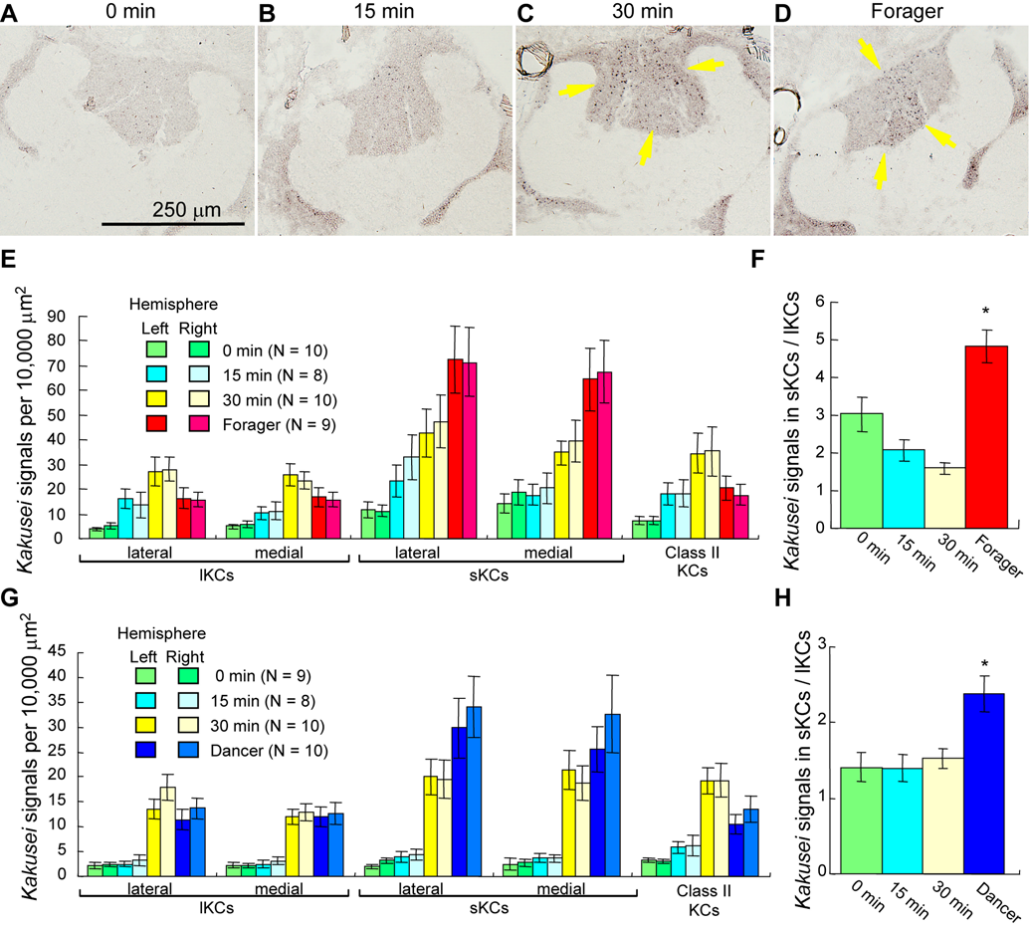
Re-orienting bees and foragers showed different *kakusei* expression patterns. (A–D) Representative *in situ* hybridization pictures of *kakusei* expression in re-orienting bees and foragers. Arrows indicate the *kakusei*-positive neurons. Note that *kakusei* expression pattern is different between the re-orienting bees and foragers. (E) Quantification of *kakusei* expression in the brains of re-orienting bees and foragers. (F) Ratio of *kakusei* density in the sKCs to lKCs. (G) Quantitative data of *kakusei* expression in re-orienting bees and dancers caught from transparent observation hives. Note the different *kakusei* expression pattern between re-orienting bees and dancers. (H) Ratio of *kakusei* density in the sKCs to lKCs. Asterisk indicates significant difference between foragers/dancers and the other groups (*, *P*<0.01; Tukey-Kramer's test after ANOVA).

Foraging behavior might involve multiple behavioral processes, such as visual, olfactory, tactile, and gustatory experiences, flying, landmark learning and memory, information processing for the dance communication, etc. We next examined whether the sKC-preferential *kakusei* expression is associated with forager-specific behavioral components or components that are common to the other behaviors, such as flying, visual experience, or landmark learning and memory. We investigated *kakusei* expression in the re-orienting bees, which were collected as the workers that fly around the hive to re-orient themselves to the hive when the location of the hive has changed. For this, we moved the hives at night with the entrance closed. The next morning, we opened the entrance for 5 min and then caught the bees flying around the hive 0, 15, and 30 min later. *Kakusei* expression was increased in the MBs in a time-dependent manner in the re-orienting bees (Figure 6A–C and E), suggesting that this neural activity is induced by re-orienting behavior. One-way ANOVA among re-orienting bees revealed that the time effect was significant for every MB neuron type (Figure 6E: *P'*s<0.0001–0.003). In contrast to sKC-preferential *kakusei* expression in the foragers, however, *kakusei* was induced in all KC types in the re-orienting bees (Figure 6A–C and E). The proportion of *kakusei*-positive cells in the sKCs compared to that in the lKCs was significantly higher in the foragers than in the re-orienting bees (Figure 6F). To exclude the possibility that the neural activity in the re-orienting bees is due to the increased light exposure when they leave the hive, a similar experiment was performed using re-orienting bees from transparent observation hives (Figure 6G and H). In this experiment, a significant time-dependent increase in *kakusei* expression was observed in every MB neuron subtype in the re-orienting bees (*P*<0.0001, one-way ANOVA), like in the re-orienting bees from the normal hives, indicating that the increased *kakusei* expression in the re-orienting bees is due to re-orienting behavior, and not merely to light-exposure (Figure 6G and H). Here, the workers that performed re-orientation flights were probably foragers, also suggesting that the differences in the *kakusei* expression pattern between the brains of re-orienting bees and foragers/dancers are due to differences in behavior, but not age. These results demonstrate that the active brain regions are different between the re-orienting bees and foragers/dancers, suggesting that the sKC-preferential *kakusei* expression in forager/dancer brains is not due to behavioral components in common with those of re-orienting bees, like visual experience, flying, or landmark learning and memory. Rather, the forager-specific behavioral components are likely to be responsible for the sKC-preferential *kakusei* expression.

## Discussion

In the present study, using novel methods to visualize neural activity in the honeybee brain, we demonstrated that neural activity in the MBs, especially the sKCs, is prominently increased in the brains of dancer and forager honeybees. In contrast, the neural activity of both the sKCs and lKCs was increased in the brains of re-orienting workers. These findings strongly suggest that sKC-preferential activity, but not mere MB-preferential activity, is associated with behavioral components that are specific to foraging flight.

The MBs are believed to be important for higher sensory integration in the insect brain [24], [25]. Partial ablation of the MBs impairs only complex olfactory learning without affecting simple olfactory learning [26], [27]. In addition, in the honeybee brain, all sensory modalities investigated (visual, olfactory, gustatory, and mechanosensory) project to the MBs [19], [20], [28]–[32]. Furthermore, foraging experience greatly influence the MB neuropile volume increase and the MB neuron outgrowth [33], [34]. Although these findings imply that the MBs are the appropriate brain regions to process complex information during foraging behavior, such as the calculation of distance and direction, there was no direct evidence whether the MB neurons are actually active in forager brains. The methods that we established in this study provide the first opportunity to investigate neural activity in the brains of naturally behaving honeybees and revealed that the MB neurons are active in the dancers and foragers, although these methods are limited to labeling only cell bodies and not the neuropile because *kakusei* encodes a non-coding RNA whose expression is confined to the nuclei.

Our study demonstrated that neural activity of the sKCs, one of the MB neuron subtypes, was prominently increased in the dancer and forager brains. Although the inputs and outputs of the sKCs have been well investigated, the role of the neural circuitry composed by the sKCs is unknown [19], [20], [28], [29], [32]. The calyx (input region) of the honeybee MB is divided into three zones: lip, collar, and basal ring. The sKCs project dendrites to the basal ring zone, whereas the lKCs project to the lip or collar zone [19]. On the other hand, all sensory modalities investigated (visual, olfactory, gustatory, and mechanosensory) project to both the basal ring and lip/collar zones, which are then relayed to both sKCs and lKCs [19], [20], [28]–[32]. Studies of the anatomy of the bee brain suggest that the basal ring zone receives multi-modal (visual, olfactory, gustatory, and mechanosensory) inputs and extensive recurrent inputs from extrinsic MB neurons, whereas the lip and collar zone receive mono-modal (olfactory and visual, respectively) inputs [19], [30], [31]. In addition, the sKCs are composed of neurons with different morphologies [19]. Thus, we speculate that the sKC-preferential neural activity observed in the forager/dancer brains reflects the complex activity within the MB neural networks required for sensory integration during the foraging flight. Recent studies suggest functional differences among the KC types based on their gene expression patterns [21], [22], [35]–[38]. For example, activation of *Amfor*, one of the genes preferentially expressed in the sKCs, affects the onset of foraging behavior [18], [38]. These findings are also consistent with our notion that the sKCs play roles in higher integration of the complex information that accompanies foraging experience.

Prominent *kakusei* expression was detected only in the forager brains, not in the follower brains (Figure 4), possibly due to the differences in their foraging experience during the hour before sampling. The followers often show reduced foraging activity before they start following the dance [7], [23]. Thus, considering the short half-life of *kakusei* expression, it is possible that this behavioral difference is reflected in the *kakusei* expression in the follower brains. In addition, there were strikingly different *kakusei* expression patterns in the MBs between the re-orienting and foraging workers. Because the re-orienting bees fly around to form spatial memory regarding their hive location [39], they have several behavioral components in common with the foragers. How then are the differences in *kakusei*-expression between re-orienting and forager/dancer bees explained? Foraging behavior is different from orienting behavior in some ways: for example, foragers need to calculate the distance and direction of food sources, memorize them in association with food information, and recall them repetitively to repeat foraging, which involves much broader and multi-modal sensory integration than just orienting [39]–[41]. Thus, it is possible that the increased activity in the sKCs in the forager brains is associated with these behavioral components and reflects such sensory integration. Alternatively, it is also possible that the sKC activity is important for both the foraging and re-orienting behaviors and the relative repression of the lKC activity is important for the foraging behaviors. If this is the case, the integration of sensory information during foraging flight might result from interactions between active sKCs and inactive lKCs.

Here we identified the transcript of novel IEG, *kakusei*, as a non-coding nuclear RNA. Although microRNA, which is expressed in response to neural activity, has been reported in vertebrates [42], [43], *kakusei* is the first example of a long non-coding nuclear RNA that shows an immediate early response to neural activity. Long nuclear RNAs regulate gene expression [44], [45], whereas microRNA post-transcriptionally regulates gene expression [46]. In general, vertebrate IEGs encode transcription factors and have roles in modulating neural functions in an activity-dependent manner [47]. Thus, *kakusei* might regulate gene expression as a long non-coding RNA to modulate neural function.

Although methods using IEGs as markers of neural activity are widely applied in vertebrates [12]–[15], no IEG has yet been reported in the insect brain. Thus, this is the first use of an IEG to identify an active brain region in the insect. Future studies examining *kakusei* expression after a well-defined experience of various sensory modalities such as gustatory, tactile, olfactory, and visual (colors, patterns, etc.) should dramatically enhance our ability to interpret the present data. Moreover, in future studies, the link between *kakusei* expression and neural activity will need to be clarified to reveal the kind of neural activity that is reflected by *kakusei* expression.

Due to limitations of the experimental methods, detailed behavioral components that induce *kakusei* expression in the sKCs remain to be examined. Nonetheless, the present study provides important insight into the neural basis of sensory integration during foraging flight, which might be related to the dance communication. It also describes a useful method for mapping active brain regions involved in behaviors of interest in the honeybee.

## Materials and Methods

### Bees

European honeybees (*A. mellifera* L.) were purchased from a local dealer and maintained at the University of Tokyo. Observation hives were made as previously described with some modification [7].

### Differential display

Worker honeybee brains were dissected out from each of 10 bees anesthetized on ice and bees awakened from ice-induced anesthesia, which showed a seizure-like phenotype. Total RNA was isolated with TRIzol (Invitrogen), treated with DNase I, and reverse transcribed with SuperScript II (Invitrogen). The differential display method was performed as described previously using a Fluorescent Differential Display Kit and LA *Taq* polymerase with a combined total of 216 primer sets (Takara) [21], [22]. Bands of interest were excised, reamplified, and subcloned into a pGEM-T vector (Promega).

### Northern blotting

Whole brain total RNA was isolated from each of 10 bees anesthetized by CO_2_ and bees awakened from CO_2_-induced anesthesia. RNA was subjected to 1% formaldehyde-agarose gel electrophoresis, transferred to a nylon membrane, and hybridized with ^32^P-labeled riboprobes. ^32^P-labeled riboprobes were synthesized by T7 polymerase with Strip-EZ^TM^ RNA Kit (Ambion) from a template containing the fragment isolated by differential display (DD fragment; from+4511 to+5159).

### cDNA cloning

To identify the whole length of *kakusei* cDNA, 5′-and 3′-rapid amplification of cDNA ends (RACE) methods were performed repeatedly using the SMART RACE cDNA Amplification Kit (Clontech).

### RT-PCR

RT-PCR experiments were performed using LA Taq (Takara) according to the manufacturer's protocol and the SMART RACE cDNA as templates. Primers were designed to amplify the regions shown in Figure 1B; (a) 5′-CACGCTCGTCGTCGTGCCTTGCTCAGATAA-3′ and 5′-TTCAGAGCACGTTGGAACTAATCTCGCG-3′, (b) 5′-ACCTTGGAACGTGAAAGCGCATTTTCGA-3′ and 5′-AACCGTGTCCTTCTGCAGACACCTGACA-3′

### Quantification of the *kakusei* transcript

The expression of *kakusei* was induced by awakening bees from CO_2_-induced anesthesia. Control bees were kept in CO_2_. Total RNA was extracted from three to five bees for each sample. Real-time RT-PCR was performed with Light Cycler-DNA master hybridization probes (Roche) according to the manufacturer's protocol, using gene-specific primers (*kakusei*; 5′-GGAAACAGGTGGTTTGATGACCATTG and 5′-CACGTTCCAAGGTTTAACGATGCG, *actin*; 5′-GAAATGGCAACTGCTGCATC and 5′-TCCACATCTGTTGGAAGGTG) and fluorescent probes (*kakusei*; fluorescein isothiocyanate (FITC) probe, 5′-CGCTGTAGTGCGTTTTCACTCGGATCGA, and LC-Red640 probe, 5′-TCCGAGGAAATCCGAGCAAAGTTCGTTC, *actin*; FITC probe, 5′-CCATGAAAATTAAGATCATCGCGCCAC, and LC-Red640 probe, 5′-CGAGAAGAAATATTCCGTATGGATTGGTG). The amount of *kakusei* transcript was normalized with that of *actin* and is shown as relative to the value of control bees at 0 min or to the whole brain. There was no significant difference in the levels of *actin* expression between control and seizure-induced bees.

### *In situ* hybridization and image analysis

*In situ* hybridization was performed as described previously with some modification [22], [48]. Frozen coronal brain sections (10 µm thick) were fixed in 4% paraformaldehyde in phosphate buffered saline, pretreated, and hybridized with digoxigen (DIG)-labeled riboprobes. The DIG-labeled riboprobes were synthesized by T7 or SP6 polymerase with a DIG labeling mix (Roche) from a template containing the fragment isolated by differential display (from+4511 to+5159). After stringent washes, DIG-labeled riboprobes were detected immunocytochemically with peroxidase-conjugated anti-DIG antibody (1:500; Roche) and TSA Biotin System (Perkin Elmer). Sense probes were used as negative controls and the signals were confirmed to be antisense probe-specific in every experiment. Micrographs of fluorescent *in situ* hybridization were taken using an IX71 confocal microscope (Olympus). SYTOX Green (Molecular Probes) was used to stain the nuclear DNA [22]. Intensity and brightness of the micrographs were processed with Photoshop software (Adobe).

For quantification, the brain regions were defined as shown in Figure 2A and B. The rostral section was defined as the section containing the AL, and the caudal section was defined as the section containing the DL. The numbers of *kakusei*-positive cells were manually counted from rostral and caudal sections. For each animal, one rostral and one caudal section were analyzed. The area of each brain region was measured using ImageJ analysis software (NIH, http://rsb.info.nih.gov/ij). The amount of signal was divided by the area and the values from two sections were averaged, when there were two data points for one individual (e.g., MB and OL neurons). Sections to be counted were randomly selected from many sections. The density of *kakusei*-positive cells was shown as a value relative to 100000 µm^2^. Micrographs were numbered and signals were counted by an investigator blind to the bee type. The number of examined bees is shown in the figure. Statistical analyses were conducted by *F*-test and Student's *t*-test using Microsoft Excel (Microsoft) or JMP (SAS) software. Multiple comparisons were performed using one-way analysis of variance (ANOVA) and Tukey-Kramer's test. Statistical comparisons were made within the same brain hemisphere. Data are shown means±standard error (SEM) throughout this paper.

## Supporting Information

Figure S1 Kakusei expression in response to light-exposure. (A–H) In situ hybridization of kakusei in the brains of dark-adapted (A, C, E, and G) or light-exposed after dark-adaptation (B, D, F, and H) bees. (C and D) Magnified view of the boxed region in panels (A) and (B), respectively. Kakusei expression was detected only in the OL neurons (B, D, and H) and MB neurons (F). Arrows indicate the kakusei-positive neurons. LA, lamina; ME, medulla; RE, retina.
(6.90 MB TIF)

Figure S2 Schematic drawings of the phototaxis experiments. First, approximately 20 foragers and 20 nurse bees were fed honey and kept separately in transparent boxes under room light conditions for 24 h (A and D: Here, only 4 bees are drawn for simplicity.). Then, a light (100 W lamp) was set in the one side of the box and kept for 30 min (B and E). During 30 min radiation, almost all of the foragers and a part of nurse bees (approximately 10 bees) moved to the light side (C and F). We therefore collected foragers that moved to the light side and nurse bees that did not move to the light side, and investigated kakusei expression.
(0.54 MB TIF)
