# Supplementary figures and images for: Increased Neural Activity of a Mushroom Body Neuron Subtype in the Brains of Forager Honeybees
Source: PLoS One. 2007 Apr 18;2(4):e371. doi: 10.1371/journal.pone.0000371 (PMC1847703; doi:10.1371/journal.pone.0000371)

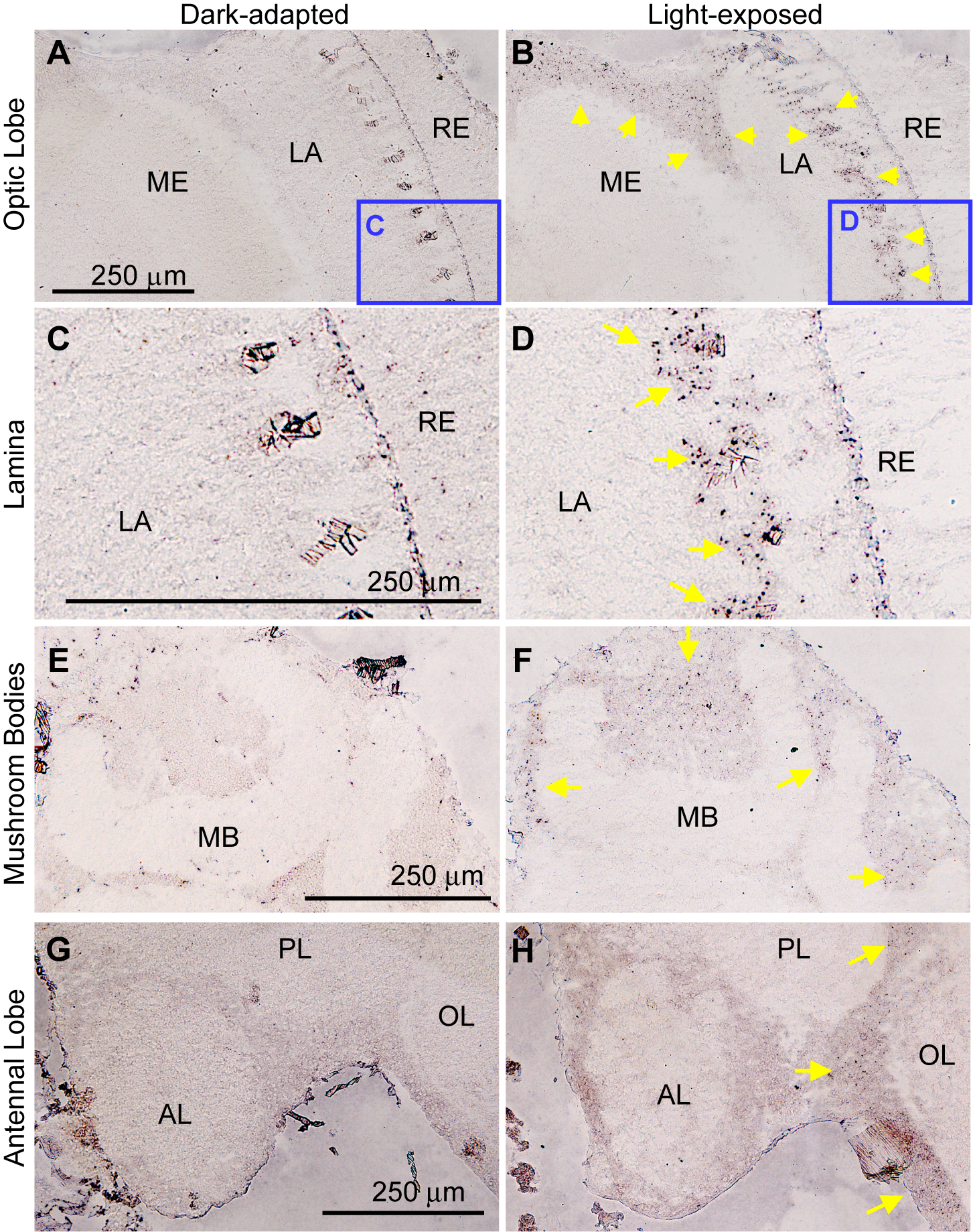

Supplement: Figure S1 — Kakusei expression in response to light-exposure. (A–H) In situ hybridization of kakusei in the brains of dark-adapted (A, C, E, and G) or light-exposed after dark-adaptation (B, D, F, and H) bees. (C and D) Magnified view of the boxed region in panels (A) and (B), respectively. Kakusei expression was detected only in the OL neurons (B, D, and H) and MB neurons (F). Arrows indicate the kakusei-positive neurons. LA, lamina; ME, medulla; RE, retina. (6.90 MB TIF) [file pone.0000371.s001.tif]

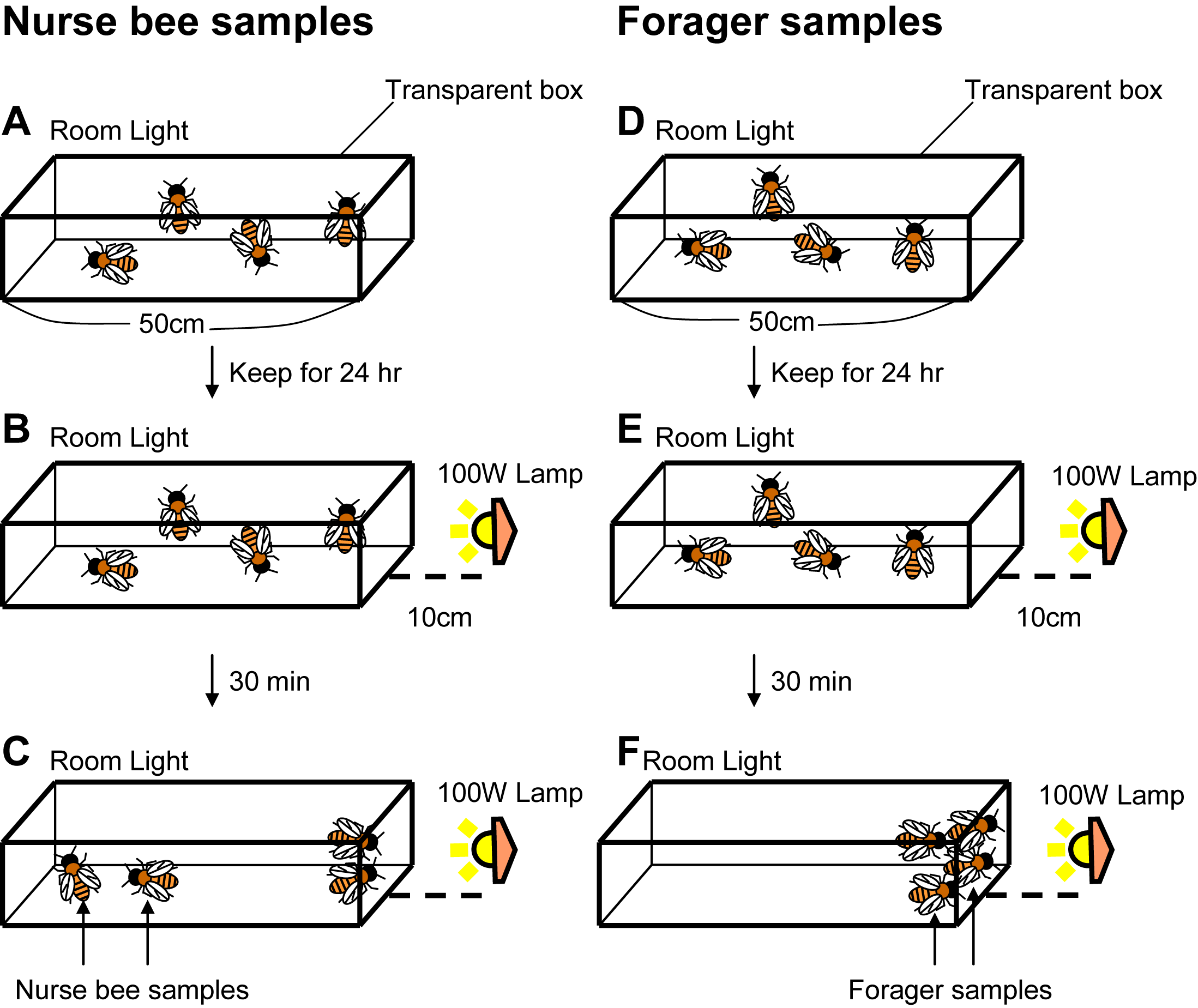

Supplement: Figure S2 — Schematic drawings of the phototaxis experiments. First, approximately 20 foragers and 20 nurse bees were fed honey and kept separately in transparent boxes under room light conditions for 24 h (A and D: Here, only 4 bees are drawn for simplicity.). Then, a light (100 W lamp) was set in the one side of the box and kept for 30 min (B and E). During 30 min radiation, almost all of the foragers and a part of nurse bees (approximately 10 bees) moved to the light side (C and F). We therefore collected foragers that moved to the light side and nurse bees that did not move to the light side, and investigated kakusei expression. (0.54 MB TIF) [file pone.0000371.s002.tif]
